# Prior COVID-19 infection and fibromyalgia criteria fulfillment: a comparative cross-sectional study

**DOI:** 10.3389/fmed.2026.1876965

**Published:** 2026-08-07

**Authors:** Mustafa Alhayali, Sami Salman, Mustafa A. Shahrori, Aya Ahmed Shimal, Marafi Jammaa Ahmed, Marwah Al-mashhadani, Zainab Shukur, Noor Alfirtusi, Ahmed Dheyaa Al-Obaidi

**Affiliations:** 1 Department of Rheumatology, Ibn Sina University of Medical and Pharmaceutical Sciences, Baghdad, Iraq; 2 Department of Medicine, College of Medicine, University of Baghdad, Baghdad, Iraq; 3 Faculty of Medicine, An-Najah National University, Nablus, Palestine; 4 College of Medicine, University of Baghdad, Baghdad, Iraq; 5 Faculty of Medicine, University of Bahri, Khartoum, Sudan; 6 Internal Medicine Department, Aldujail Hospital, Salahaldin, Iraq; 7 Department of Internal Medicine, West Anaheim Medical Center, Anaheim, CA, United States; 8 Heart and Vascular Consultants DMC, Associate Retinal Consultants, Royal Oak, MI, United States; 9 College of Medicine, University of Baghdad, Baghdad, Iraq

**Keywords:** American College of Rheumatology criteria, chronic widespread pain, comparative cross-sectional study, COVID-19, fibromyalgia, prior COVID-19 infection, SARS-CoV-2

## Abstract

**Background:**

Fibromyalgia (FM) is a chronic widespread pain syndrome frequently accompanied by fatigue, cognitive dysfunction, psychological symptoms, and multiple somatic symptoms. Coronavirus disease 2019 (COVID-19) has affected millions worldwide; however, its long-term sequelae remain incompletely understood.

**Objective:**

This study aimed to investigate whether prior COVID-19 infection was associated with a higher prevalence of fibromyalgia and to identify demographic and clinical factors associated with fibromyalgia in the study population.

**Subjects and methods:**

This comparative cross-sectional study included 250 individuals with PCR-confirmed COVID-19 at least 3 months before assessment and 250 participants without a known history of confirmed COVID-19 infection. Data were collected at a single time point using a structured questionnaire and interview. Fibromyalgia status was defined as fulfillment of the modified 2016 American College of Rheumatology criteria.

**Results:**

Fibromyalgia criteria were fulfilled by 60/250 participants (24.0%) with prior COVID-19 infection compared with 35/250 participants (14.0%) in the comparison group. In crude analysis, prior COVID-19 infection was associated with higher odds of fibromyalgia criteria fulfillment (OR = 1.94, 95% CI: 1.22–3.07, *p* = 0.004). After adjustment for age group, sex, educational level, occupation, smoking status, and chronic disease, prior COVID-19 infection remained significantly associated with higher odds of fibromyalgia criteria fulfillment (adjusted OR = 2.18, 95% CI: 1.33–3.58, *p* = 0.002).

**Conclusion:**

Prior COVID-19 infection remained associated with higher odds of modified 2016 ACR fibromyalgia criteria fulfillment. Female sex and non-employment were also associated with fibromyalgia criteria fulfillment, highlighting the need for clinical awareness and early recognition of fibromyalgia-like symptoms among individuals with a history of COVID-19 infection.

## Highlights

- Fibromyalgia criteria fulfillment was more prevalent among participants with prior COVID-19 infection than in the comparison group.
- Prior COVID-19 infection remained independently associated with higher odds of fibromyalgia criteria fulfillment after adjustment for demographic and clinical variables.
- Female sex and non-employment were also independently associated with fibromyalgia criteria fulfillment.

## 1 Introduction

Fibromyalgia (FM) is characterized by chronic widespread musculoskeletal pain accompanied by fatigue, nonrestorative sleep, cognitive impairment, depression, and anxiety (1, 2). It is a persistent, potentially disabling condition with substantial impact on quality of life (3). Population-based studies estimate a prevalence of approximately 2–5% in the general population and affect more females than males (4–6). Regional estimates vary; a systematic review and meta-analysis from Saudi Arabia reported a pooled fibromyalgia prevalence of 13.4% (25).

Fibromyalgia has a multifactorial etiology characterized by complex interactions between genetic susceptibility, epigenetic modifications, neurobiological alterations, and environmental triggers such as physical trauma, infections, and psychosocial stressors (3, 7). Infectious agents have been associated with the onset of fibromyalgia, particularly following prolonged infectious illness. Recent observations of fibromyalgia-like manifestations after SARS-CoV-2 infection further support a possible post-infectious contribution to disease pathogenesis (8–12). Several recent studies have brought to light many similarities between fibromyalgia and long COVID in terms of both the symptom profile, including widespread pain, fatigue, sleep disorders, cognitive symptoms, and autonomic dysfunction, and the possible disease pathophysiology concerning a post-infection origin, which can be regarded as a link between these two entities (8, 9). Evidence suggests that several cases of fibromyalgia following SARS-CoV-2 infection have been reported (10). Some theories include sustained immune response, neuroinflammation, autonomic dysfunction, and central sensitization as possible triggers of disease pathogenesis (3). Consequently, SARS-CoV-2 infection has been suggested as a possible trigger for the development of fibromyalgia in susceptible individuals (9).

Since there is significant overlap between post-COVID-19 symptoms and those of fibromyalgia, in addition to increasing knowledge on the potential association between SARS-CoV-2 infection and fibromyalgia, exploring the connection between COVID-19 and FM is medically important. However, data about the prevalence of FM after COVID-19 infection and the associated demographic and clinical factors are currently insufficient, especially with regard to Middle Eastern populations. This study was thus carried out to investigate whether prior COVID-19 infection was associated with a higher prevalence of FM and to identify factors associated with FM among the study participants.

## 2 Materials and methods

### 2.1 Study design and population

This comparative cross-sectional study was conducted between January 2024 and January 2025. A total of 500 adult participants were recruited consecutively and classified according to prior COVID-19 infection status. The sample size was determined by the number of eligible participants available during the study period who met the predefined inclusion and exclusion criteria. Both groups were recruited during the same study period from the same healthcare setting.

The prior COVID-19 group included 250 adults with a documented history of SARS-CoV-2 infection confirmed by nasopharyngeal swab polymerase chain reaction (PCR). Participants in this group were identified through outpatient follow-up clinics and community attendees and had recovered from the acute infection at least 3 months before assessment.

The comparison group included 250 adults recruited concurrently from the rheumatology outpatient clinic of the same institution who had no known history of confirmed COVID-19 infection. Recruitment from the same healthcare environment was intended to improve comparability regarding healthcare access and geographic catchment area between groups.

The comparison group was selected from rheumatology outpatient attendees because they were available during the same recruitment period and could undergo the same structured fibromyalgia assessment. Their common presenting complaints or working diagnoses included localized musculoskeletal pain, osteoarthritis, mechanical back pain, periarticular disorders, regional soft-tissue rheumatism, and follow-up of stable non-inflammatory musculoskeletal conditions. Participants presenting primarily with chronic widespread pain or with a previous diagnosis of fibromyalgia were excluded, as described in the eligibility criteria.

For all participants, a detailed clinical history was obtained, including demographic characteristics such as age, sex, marital status, educational level, occupation, smoking status, alcohol intake, and relevant medical history. Participants were subsequently assessed for fulfillment of the 2016 revision of the 2010/2011 American College of Rheumatology fibromyalgia criteria.

### 2.2 Eligibility criteria

Eligible participants were adults aged 18 years or older. For the prior COVID-19 group, participants were required to have a documented history of COVID-19 infection confirmed by PCR and to have recovered from the infection at least 3 months before enrollment.

Participants in the comparison group were required to have no known history of confirmed COVID-19 infection. Because systematic PCR or serological testing was not performed at enrollment, this group was not considered confirmed free of prior SARS-CoV-2 infection and is described as participants without a known history of confirmed COVID-19 infection.

Individuals younger than 18 years were excluded. Participants who reported symptoms suggestive of COVID-19 infection or contact with confirmed cases without PCR confirmation were not included in the prior COVID-19 group. Additional exclusion criteria included a previous diagnosis of inflammatory arthritis or connective tissue disease, including rheumatoid arthritis, systemic lupus erythematosus, Sjögren’s syndrome, systemic sclerosis, mixed connective tissue disease, spondyloarthropathies, and systemic vasculitis. Participants with a history of malignancy, thyroid disease, osteomalacia, or neurological disorders such as multiple sclerosis or myasthenia gravis were also excluded. Participants with a known previous diagnosis of fibromyalgia or chronic widespread pain syndrome were excluded based on self-reported history and review of available medical records when available. Pregnant women and individuals who declined participation were excluded. Participants with known psychiatric disorders were excluded based on self-reported medical history and review of available medical records. No formal psychiatric screening instruments were administered during recruitment.

### 2.3 Fibromyalgia assessment

Fibromyalgia criteria fulfillment was assessed using an Arabic-language version of the modified 2016 American College of Rheumatology fibromyalgia criteria questionnaire, administered through a structured interview (13). An Arabic version of the Fibromyalgia Survey Questionnaire has been validated in Arabic-speaking populations (14). The assessment included calculation of the Widespread Pain Index and Symptom Severity Scale.

The Widespread Pain Index was calculated according to the number of painful body areas reported by the participant. The Symptom Severity Scale assessed fatigue, unrefreshing sleep, cognitive symptoms, and associated somatic symptoms. Participants were classified as fulfilling the modified 2016 ACR fibromyalgia criteria if they met the required WPI and SSS thresholds, had generalized pain involving at least four of five body regions, and reported symptoms at a similar level for at least 3 months. The WPI ranges from 0 to 19 and the SSS ranges from 0 to 12. Participants fulfilled the symptom-score criterion if WPI was ≥7 with SSS ≥5, or if WPI was 4–6 with SSS ≥9.

Pain distribution and symptom duration were specifically verified during the interview. Fibromyalgia status in this study was based on fulfillment of the modified 2016 ACR criteria using the structured questionnaire and interview process. No separate rheumatologist-confirmed clinical diagnosis was required for study classification; therefore, the outcome is described as fulfillment of the modified 2016 ACR fibromyalgia criteria rather than a separate clinically confirmed diagnosis of fibromyalgia.

Data collection was performed by trained physicians or research personnel who received standardized instruction regarding questionnaire administration and application of the ACR criteria. Assessors were aware of participants’ COVID-19 status; therefore, lack of assessor blinding was considered a potential source of assessment bias.

### 2.4 Ethical considerations

Approval from the relevant health authority and Institutional Review Board was obtained before the initiation of data collection. Written informed consent was waived because the study involved minimal risk and anonymous data collection. Verbal informed consent was obtained and documented by the research team before participant enrollment, after a full explanation of the study objectives and voluntary participation. Confidentiality and anonymity of all collected information were maintained throughout the study, and the data were used exclusively for research purposes.

### 2.5 Statistical analysis

Data were entered into Microsoft Excel 2016 and analyzed using the Statistical Package for the Social Sciences (SPSS), version 24. Categorical variables were summarized as frequencies and percentages. Comparisons between categorical variables were performed using Pearson’s chi-square test when test assumptions were met. Expected cell counts were reviewed before selecting the appropriate test. Fisher’s exact test was used for variables with small expected cell counts, particularly sparse comparisons such as alcohol intake, asthma, cerebrovascular accident, and ischemic heart disease.

Crude odds ratios with 95% confidence intervals were calculated to estimate the unadjusted association between prior COVID-19 infection and fulfillment of the modified 2016 ACR fibromyalgia criteria. Because the outcome was not rare, the absolute prevalence difference and crude prevalence ratio were also calculated to provide prevalence-based measures of association.

To account for potential confounding, multivariable binary logistic regression was performed with fulfillment of the modified 2016 ACR fibromyalgia criteria as the dependent variable. Prior COVID-19 infection was included as the main exposure variable. The model was adjusted for age group, sex, educational level, occupation, smoking status, and the presence of any chronic disease. These variables were selected *a priori* based on clinical relevance and their potential association with both prior COVID-19 status and fibromyalgia criteria fulfillment. Adjusted odds ratios with 95% confidence intervals were reported.

Missing data were reviewed before analysis. Participants with missing data for variables required in the primary analysis were excluded from the relevant analysis. Multicollinearity among variables included in the regression model was assessed before final model interpretation. Model performance and calibration were evaluated using standard logistic regression diagnostics.

Because the outcome was not rare, a modified Poisson regression model with robust standard errors was additionally performed to estimate adjusted prevalence ratios. The model included the same covariates as the primary logistic regression model. Because employment status may be affected by fibromyalgia-related symptoms and functional impairment, a sensitivity analysis excluding occupation from the adjusted model was also performed. Univariate analyses were considered exploratory. No formal correction for multiple comparisons was applied because the primary analysis was the multivariable regression model. A two-sided *p*-value of less than 0.05 was considered statistically significant.

## 3 Results

**Figure 1:**
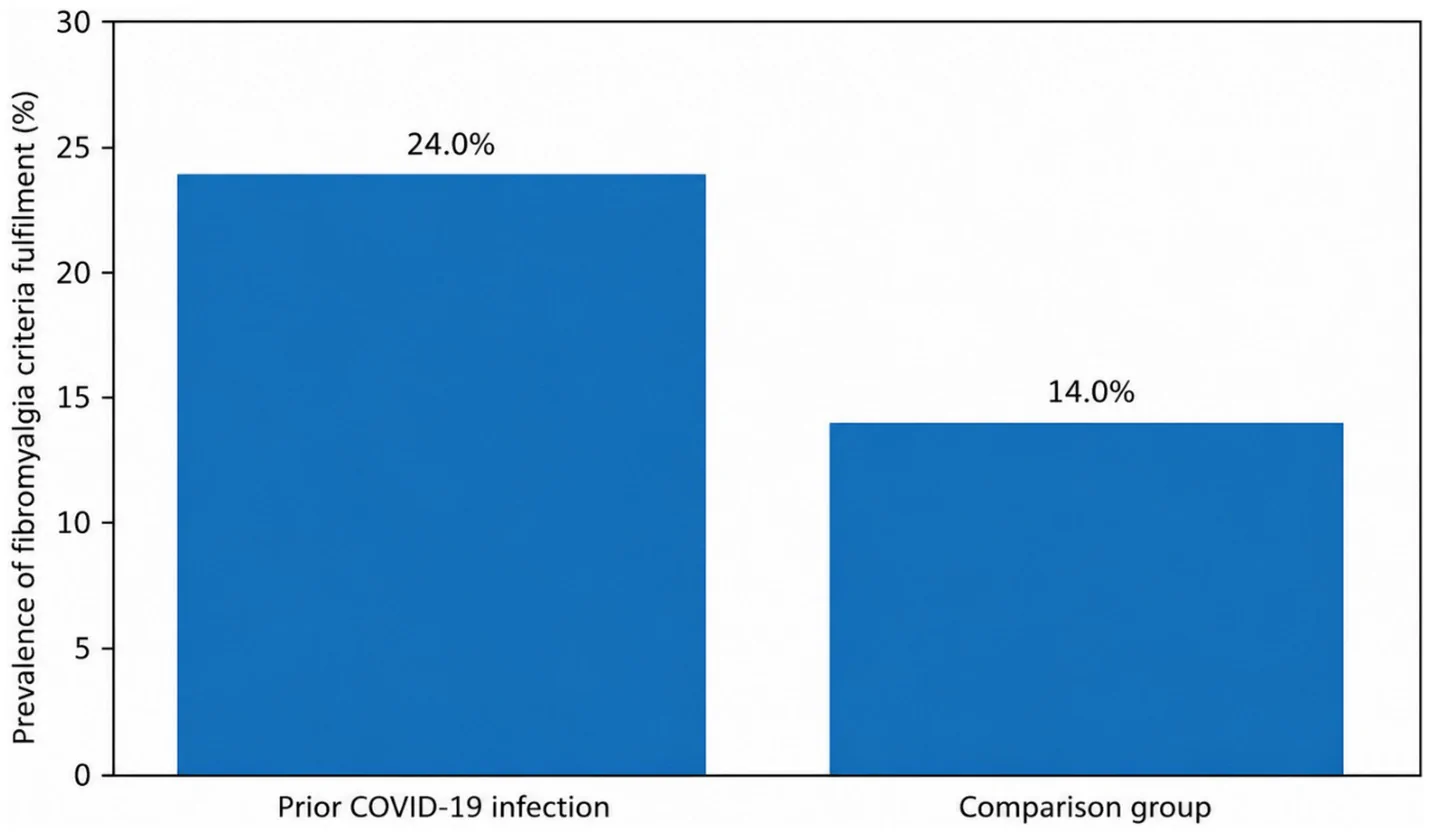
Prevalence of fibromyalgia criteria fulfillment according to prior COVID-19 infection status.

This comparative cross-sectional study included 500 participants: 250 participants with prior PCR-confirmed COVID-19 infection and 250 participants in the comparison group without a known history of confirmed COVID-19 infection. Fibromyalgia criteria were fulfilled by 60/250 participants with prior COVID-19 infection and 35/250 participants in the comparison group. The prevalence of fibromyalgia criteria fulfillment was higher in the prior COVID-19 group than in the comparison group (24.0% vs. 14.0%). In crude analysis, prior COVID-19 infection was associated with higher odds of fibromyalgia criteria fulfillment (crude OR = 1.94, 95% CI: 1.22–3.07, *p* = 0.004). The absolute prevalence difference was 10.0 percentage points, and the crude prevalence ratio was 1.71 (Table 1; Figure 1).

**Table 1:** Prevalence of fibromyalgia criteria fulfillment according to prior COVID-19 infection status.

Group	Fibromyalgia *n* (%)	No fibromyalgia *n* (%)	Crude OR	95% CI	*p*-value
Prior COVID-19 infection group	60 (24.0%)	190 (76.0%)	1.94	1.22–3.07	0.004
Comparison group	35 (14.0%)	215 (86.0%)	Reference	–	–

Baseline characteristics according to prior COVID-19 infection status are presented in Table 2. The two groups were generally comparable in marital status, smoking status, alcohol intake, asthma, diabetes mellitus, hypertension, cerebrovascular accident, ischemic heart disease, and the presence of any chronic disease. However, sex distribution differed significantly between groups, with a higher proportion of females in the comparison group than in the prior COVID-19 group (68.0% vs. 55.6%, *p* = 0.004). Educational level also differed significantly between groups (*p* = 0.037), with a higher proportion of university-level education in the prior COVID-19 group. Age distribution, occupation, and ischemic heart disease showed borderline differences but did not reach conventional statistical significance. These variables were considered in the adjusted regression analysis where appropriate.

**Table 2:** Baseline characteristics according to prior COVID-19 infection status.

Variable	Category	Prior COVID-19 group *n* (%)	Comparison group *n* (%)	*p*-value
Age group	18–24 years	11 (4.4%)	19 (7.6%)	0.074
	25–34 years	71 (28.4%)	50 (20.0%)	
	35–44 years	44 (17.6%)	40 (16.0%)	
	45–54 years	42 (16.8%)	58 (23.2%)	
	≥55 years	82 (32.8%)	83 (33.2%)	
Sex	Male	111 (44.4%)	80 (32.0%)	0.004
	Female	139 (55.6%)	170 (68.0%)	
Marital status	Single	42 (16.8%)	43 (17.2%)	0.579
	Married	174 (69.6%)	165 (66.0%)	
	Other	34 (13.6%)	42 (16.8%)	
Smoking	Smoker	62 (24.8%)	59 (23.6%)	0.754
	Non-smoker	188 (75.2%)	191 (76.4%)	
Alcohol intake	Yes	10 (4.0%)	6 (2.4%)	0.447*
	No	240 (96.0%)	244 (97.6%)	
Education	Primary or lower	25 (10.0%)	43 (17.2%)	0.037
	Secondary	75 (30.0%)	79 (31.6%)	
	University or higher	150 (60.0%)	128 (51.2%)	
Occupation	Employed	159 (63.6%)	138 (55.2%)	0.056
	Non-employed	91 (36.4%)	112 (44.8%)	
Asthma	Yes	10 (4.0%)	12 (4.8%)	0.828*
	No	240 (96.0%)	238 (95.2%)	
Diabetes mellitus	Yes	25 (10.0%)	35 (14.0%)	0.169
	No	225 (90.0%)	215 (86.0%)	
Hypertension	Yes	57 (22.8%)	48 (19.2%)	0.323
	No	193 (77.2%)	202 (80.8%)	
Cerebrovascular accident	Yes	0 (0.0%)	3 (1.2%)	0.248*
	No	250 (100.0%)	247 (98.8%)	
Ischemic heart disease	Yes	3 (1.2%)	11 (4.4%)	0.054*
	No	247 (98.8%)	239 (95.6%)	
Any chronic disease	Yes	65 (26.0%)	59 (23.6%)	0.534
	No	185 (74.0%)	191 (76.4%)	

Values are presented as *n* (%). Percentages were calculated within prior COVID-19 infection status groups. *P*-values were calculated using Pearson’s chi-square test unless otherwise indicated. *Fisher’s exact test was used for sparse 2×2 comparisons. Any chronic disease was defined as the presence of diabetes mellitus, hypertension, ischemic heart disease, or cerebrovascular accident.

Participant characteristics stratified by fibromyalgia status are presented in Table 3. In univariate analysis, significant differences were observed between participants with and without fibromyalgia in age group, sex, educational level, and occupation. Participants with fibromyalgia were more frequently female than those without fibromyalgia (80.0% vs. 57.5%, *p* < 0.001) and were more commonly non-employed (52.6% vs. 37.8%, *p* = 0.008). Age distribution also differed significantly between groups (*p* = 0.040), with a lower proportion of participants aged ≥55 years among those with fibromyalgia. Educational level showed a significant association with fibromyalgia status (*p* = 0.032). In contrast, marital status, smoking status, alcohol intake, asthma, diabetes mellitus, hypertension, cerebrovascular accident, ischemic heart disease, and the presence of any chronic disease were not significantly associated with fibromyalgia.

**Table 3:** Participant characteristics according to fibromyalgia status.

Variable	Category	Fibromyalgia *n* (%)	No fibromyalgia *n* (%)	*p*-value
Age group	18–24 years	5 (5.3%)	25 (6.2%)	0.040
	25–34 years	27 (28.4%)	94 (23.2%)	
	35–44 years	19 (20.0%)	65 (16.0%)	
	45–54 years	25 (26.3%)	75 (18.5%)	
	≥55 years	19 (20.0%)	146 (36.0%)	
Sex	Male	19 (20.0%)	172 (42.5%)	<0.001
	Female	76 (80.0%)	233 (57.5%)	
Marital status	Single	17 (17.9%)	68 (16.8%)	0.658
	Married	61 (64.2%)	278 (68.6%)	
	Other	17 (17.9%)	59 (14.6%)	
Smoking	Smoker	22 (23.2%)	99 (24.4%)	0.792
	Non-smoker	73 (76.8%)	306 (75.6%)	
Alcohol intake	Yes	4 (4.2%)	12 (3.0%)	0.520*
	No	91 (95.8%)	393 (97.0%)	
Education	Primary or lower	8 (8.4%)	60 (14.8%)	0.032
	Secondary	39 (41.1%)	115 (28.4%)	
	University or higher	48 (50.5%)	230 (56.8%)	
Occupation	Employed	45 (47.4%)	252 (62.2%)	0.008
	Non-employed	50 (52.6%)	153 (37.8%)	
Asthma	Yes	7 (7.4%)	15 (3.7%)	0.159*
	No	88 (92.6%)	390 (96.3%)	
Diabetes mellitus	Yes	15 (15.8%)	45 (11.1%)	0.207
	No	80 (84.2%)	360 (88.9%)	
Hypertension	Yes	21 (22.1%)	84 (20.7%)	0.769
	No	74 (77.9%)	321 (79.3%)	
Cerebrovascular accident	Yes	0 (0.0%)	3 (0.7%)	1.000*
	No	95 (100.0%)	402 (99.3%)	
Ischemic heart disease	Yes	1 (1.1%)	13 (3.2%)	0.486*
	No	94 (98.9%)	392 (96.8%)	
Any chronic disease	Yes	26 (27.4%)	98 (24.2%)	0.520
	No	69 (72.6%)	307 (75.8%)	

Values are presented as *n* (%). Percentages were calculated within fibromyalgia status groups. *p*-values were calculated using Pearson’s chi-square test unless otherwise indicated. *Fisher’s exact test was used because of small expected cell counts. Any chronic disease was defined as the presence of diabetes mellitus, hypertension, ischemic heart disease, or cerebrovascular accident.

Multivariable logistic regression was performed to assess factors associated with fibromyalgia criteria fulfillment after adjustment for potential confounders. The model included prior COVID-19 infection, age group, sex, educational level, occupation, smoking status, and the presence of any chronic disease. After adjustment, prior COVID-19 infection remained significantly associated with higher odds of fibromyalgia criteria fulfillment (adjusted OR = 2.18, 95% CI: 1.33–3.58, *p* = 0.002). Female sex was also independently associated with fibromyalgia (adjusted OR = 4.13, 95% CI: 2.05–8.29, *p* < 0.001). Compared with participants aged ≥55 years, those aged 25–34, 35–44, and 45–54 years had significantly higher adjusted odds of fibromyalgia. Non-employment and smoking were also associated with higher adjusted odds of fibromyalgia. The presence of any chronic disease showed a modest association with fibromyalgia in the adjusted model (adjusted OR = 1.92, 95% CI: 1.00–3.69, *p* = 0.049), although this finding should be interpreted cautiously given the marginal level of statistical significance. Educational level was not independently associated with fibromyalgia after adjustment.

Because fibromyalgia criteria fulfillment was relatively common, modified Poisson regression with robust standard errors was performed to estimate prevalence ratios. In this model, prior COVID-19 infection remained associated with a higher prevalence of fibromyalgia criteria fulfillment after adjustment for age group, sex, educational level, occupation, smoking status, and chronic disease (adjusted prevalence ratio = 1.73, 95% CI: 1.21–2.49, *p* = 0.003). In a sensitivity logistic regression model excluding occupation, the association between prior COVID-19 infection and fibromyalgia criteria fulfillment remained essentially unchanged (adjusted OR = 2.17, 95% CI: 1.33–3.56, *p* = 0.002; Table 4).

**Table 4:** Multivariable logistic regression for factors associated with fibromyalgia criteria fulfillment.

Variable	Category	Adjusted OR	95% CI	*p*-value
Prior COVID-19 infection	No	Reference	–	–
	Yes	2.18	1.33–3.58	0.002
Age group	≥55 years	Reference	–	–
	18–24 years	1.48	0.45–4.82	0.516
	25–34 years	4.35	1.90–9.94	<0.001
	35–44 years	2.72	1.21–6.12	0.015
	45–54 years	2.80	1.32–5.93	0.007
Sex	Male	Reference	–	–
	Female	4.13	2.05–8.29	<0.001
Education	University or higher	Reference	–	–
	Primary or lower	0.48	0.19–1.24	0.131
	Secondary	1.35	0.74–2.44	0.326
Occupation	Employed	Reference	–	–
	Non-employed	1.98	1.07–3.64	0.029
Smoking status	Non-smoker	Reference	–	–
	Smoker	2.01	1.01–3.99	0.046
Any chronic disease	No	Reference	–	–
	Yes	1.92	1.00–3.69	0.049

Adjusted OR: adjusted odds ratio; CI: confidence interval. The dependent variable was fulfillment of the modified 2016 ACR fibromyalgia criteria. The model was adjusted for prior COVID-19 infection, age group, sex, educational level, occupation, smoking status, and the presence of any chronic disease. Any chronic disease was defined as the presence of diabetes mellitus, hypertension, ischemic heart disease, or cerebrovascular accident.

Results of the prevalence-based and sensitivity analyses are presented in Table 5.

**Table 5:** Prevalence-based and sensitivity analyses for the association between prior COVID-19 infection and fibromyalgia criteria fulfillment.

Analysis	Effect measure	Estimate	95% CI	*p*-value
Crude prevalence comparison	Crude prevalence ratio	1.71	1.17–2.50	0.005
Modified Poisson regression with robust standard errors	Adjusted prevalence ratio	1.73	1.21–2.49	0.003
Sensitivity logistic regression excluding occupation	Adjusted odds ratio	2.17	1.33–3.56	0.002

All estimates compare the prior COVID-19 infection group with the comparison group without a known history of confirmed COVID-19 infection. The modified Poisson model was adjusted for age group, sex, educational level, occupation, smoking status, and chronic disease. The sensitivity logistic regression model excluded occupation.

## 4 Discussion

In this comparative cross-sectional study, fulfillment of the modified 2016 ACR fibromyalgia criteria was more frequent among participants with prior PCR-confirmed COVID-19 infection than among participants without a known history of confirmed COVID-19 infection. Fibromyalgia criteria were fulfilled by 24.0% of participants in the prior COVID-19 group compared with 14.0% in the comparison group, corresponding to an absolute prevalence difference of 10.0 percentage points and a crude prevalence ratio of 1.71. Prior COVID-19 infection also remained associated with higher odds of fibromyalgia criteria fulfillment after adjustment for age group, sex, educational level, occupation, smoking status, and chronic disease. The association was also supported by prevalence-based modeling, which showed that prior COVID-19 infection was associated with a 73% higher adjusted prevalence of fibromyalgia criteria fulfillment.

These findings are consistent with previous literature describing overlap between persistent symptoms after SARS-CoV-2 infection and fibromyalgia-related symptom domains, including widespread pain, fatigue, sleep disturbance, cognitive symptoms, and somatic complaints (8, 10–12, 15, 16). Several mechanisms have been proposed to explain fibromyalgia-like symptoms after infection, including persistent immune activation, neuroinflammation, autonomic dysfunction, and central sensitization (3, 7–9). However, the present study cannot determine whether SARS-CoV-2 infection directly caused fibromyalgia criteria fulfillment, because fibromyalgia status was assessed at a single time point after infection and pre-infection symptom status could not be fully verified.

It is also important to distinguish infection-specific explanations from broader pandemic-related influences. Pandemic restrictions, social isolation, financial stress, occupational disruption, reduced physical activity, psychological distress, and limited access to healthcare may all contribute to chronic pain and fibromyalgia-like symptoms (17–20). These factors likely affected both the prior COVID-19 and comparison groups. Therefore, general pandemic-related stress alone may not fully explain the observed between-group difference, although it may have contributed to symptom burden in both groups and remains an important potential confounder.

The prevalence of fibromyalgia criteria fulfillment in the comparison group was also relatively high compared with general population estimates. This may be explained partly by the clinic-based nature of the comparison group, as participants were recruited from a rheumatology outpatient setting rather than from the general population. Although individuals presenting primarily with chronic widespread pain or previously diagnosed fibromyalgia were excluded, some comparison participants had localized musculoskeletal complaints or other non-inflammatory rheumatologic conditions that may have contributed to pain, fatigue, or somatic symptom reporting. This should be considered when interpreting the absolute prevalence estimates and limits direct generalization to community populations.

Female sex was independently associated with fibromyalgia criteria fulfillment, which is consistent with previous studies showing a female predominance among individuals with fibromyalgia (21). Non-employment was also associated with higher odds of fibromyalgia criteria fulfillment. However, this finding should be interpreted cautiously because the cross-sectional design does not establish directionality. Non-employment may contribute to symptoms through socioeconomic stress, reduced activity, or limited access to care, but reverse causality is also plausible because pain, fatigue, cognitive symptoms, and functional impairment may reduce work capacity and employment participation (22–24).

Smoking and chronic disease showed only borderline adjusted associations and should be interpreted as exploratory findings. The association with chronic disease was marginal, and the chronic disease variable combined heterogeneous conditions. Similarly, the smoking association requires cautious interpretation because residual confounding and unmeasured behavioral or socioeconomic factors may have influenced the result.

Overall, the findings suggest an association between prior COVID-19 infection and later fulfillment of fibromyalgia criteria in this study population. However, given the observational and cross-sectional nature of the study, the results should be interpreted as hypothesis-generating. Prospective studies with pre-infection symptom assessment, systematic COVID-19 exposure verification in comparison groups, and measurement of psychological, sleep-related, metabolic, and socioeconomic factors are needed to clarify whether SARS-CoV-2 infection independently contributes to fibromyalgia-like symptom development.

### 4.1 Limitations and future directions

Several limitations should be considered when interpreting the findings of this study. First, because of the comparative cross-sectional design, fibromyalgia criteria fulfillment was assessed at a single time point after COVID-19 infection. Therefore, temporal relationships cannot be firmly established, and causality cannot be inferred. Although prior COVID-19 infection was associated with higher odds of fibromyalgia criteria fulfillment, the study cannot confirm that COVID-19 directly caused new-onset fibromyalgia.

Second, pre-existing fibromyalgia symptoms or chronic widespread pain before COVID-19 infection could not be fully excluded. Participants with a known previous diagnosis of fibromyalgia or chronic widespread pain syndrome were excluded based on self-reported history and review of available medical records when available, but undiagnosed or undocumented pre-existing symptoms may have been present in some participants.

Third, the exact interval between COVID-19 infection and fibromyalgia assessment was not available for all participants. Therefore, the relationship between time since infection and fibromyalgia criteria fulfillment could not be evaluated. Data on reinfection and vaccination status were also not available, limiting assessment of their possible influence on persistent symptoms.

Fourth, the severity of acute COVID-19 could not be assessed because objective clinical data such as oxygen saturation, need for hospitalization, blood gas analysis, inflammatory markers, and radiological findings were not consistently available. Therefore, the relationship between COVID-19 severity and fibromyalgia criteria fulfillment could not be examined.

Fifth, exposure misclassification may have occurred in the comparison group. Participants in the comparison group had no known history of confirmed COVID-19 infection, but systematic PCR or serological testing was not performed at enrollment. As a result, previous asymptomatic or unrecognized SARS-CoV-2 infection could not be excluded.

Sixth, selection bias may have occurred because participants were recruited from outpatient settings, and the comparison group consisted of rheumatology outpatient clinic attendees rather than a general population sample. Although participants presenting primarily with chronic widespread pain or fibromyalgia-like syndromes were excluded, some comparison participants had localized musculoskeletal complaints or non-inflammatory rheumatologic conditions that may have influenced pain or somatic symptom reporting. This may partly explain the relatively high prevalence of fibromyalgia criteria fulfillment in the comparison group.

Seventh, fibromyalgia status was based on fulfillment of the modified 2016 ACR criteria using a structured questionnaire and interview. No separate rheumatologist-confirmed clinical diagnosis was required for study classification. Therefore, the outcome should be interpreted as fibromyalgia criteria fulfillment rather than independently confirmed clinical fibromyalgia. In addition, assessors were aware of participants’ COVID-19 status, so assessment bias cannot be excluded.

Eighth, some data were based on self-report and clinical history, which may introduce recall bias. Psychiatric disorders were excluded based on self-reported history and available medical records, but formal psychiatric screening instruments were not administered. Depression, anxiety, sleep disturbance, somatic symptom burden, body mass index, physical activity, socioeconomic status, medication use, and healthcare-seeking behavior were not formally measured, although these factors may influence fibromyalgia symptoms and may act as residual confounders.

Ninth, although the multivariable logistic regression model adjusted for several clinically relevant covariates, residual confounding remains possible. Employment status should also be interpreted cautiously because it may be affected by fibromyalgia-related symptoms and functional impairment rather than acting only as a baseline confounder. Although sensitivity analysis excluding occupation and modified Poisson regression were performed, residual confounding remains possible because several clinically relevant variables were not measured.

Finally, the study was conducted in a single healthcare setting with a moderate sample size, which may limit generalizability to other populations. Future prospective longitudinal studies with larger and more diverse samples are needed to confirm these findings, document pre-infection symptom status, verify COVID-19 exposure status in comparison groups, assess COVID-19 severity, vaccination, reinfection, time since infection, psychological and sleep-related factors, and evaluate whether prior SARS-CoV-2 infection independently contributes to fibromyalgia criteria fulfillment.

## 5 Conclusion

In this comparative cross-sectional study, participants with prior PCR-confirmed COVID-19 infection had a higher prevalence of modified 2016 ACR fibromyalgia criteria fulfillment than participants without a known history of confirmed COVID-19 infection. This association remained significant after adjustment for age group, sex, educational level, occupation, smoking status, and chronic disease. Female sex and non-employment were also associated with higher odds of fibromyalgia criteria fulfillment, although the employment finding should be interpreted cautiously because reverse causality is possible. Smoking and chronic disease showed borderline associations and should be considered exploratory.

Given the observational design, clinic-based comparison group, lack of systematic COVID-19 testing in the comparison group, and potential residual confounding, these findings indicate an association rather than a causal relationship. Further prospective studies are needed to clarify the temporal relationship between SARS-CoV-2 infection and fibromyalgia-like symptom burden.

## Data Availability

The raw data supporting the conclusions of this article will be made available by the authors, without undue reservation.
